# Risk factors for caesarean delivery and fetal macrosomia among women with gestational diabetes in Nyeri County, Kenya: a cross-section study

**DOI:** 10.11604/pamj.2022.41.322.29734

**Published:** 2022-04-20

**Authors:** Peter Kimani Njogu, Eliphas Gitonga Makunyi, Joseph Musau

**Affiliations:** 1 Department of Population, Community Health and Reproductive Health, Kenyatta University, Nairobi, Kenya,; 2 Department of Clinical Pharmacy and Pharmacology, Kenyatta University, Nairobi, Kenya

**Keywords:** Gestational diabetes mellitus, caesarean delivery, fetal macrosomia

## Abstract

**Introduction:**

gestational diabetes mellitus is an emerging global public health threat due to adverse health outcomes. This study aimed to determine the risk factors for caesarean delivery and macrosomia among women with gestational diabetes in Nyeri County, Kenya.

**Methods:**

this study used a cross-section design. Randomly, 152 women with gestational diabetes and attending antenatal clinics and maternity were enrolled in this study. Data was collected using a questionnaire upon consent. Data were subjected to binary logistic regression and binomial multiple logistic regression.

**Results:**

the mean age of the women with gestational diabetes was 30.86 (SD 5.81) years. Among women with gestational diabetes, a proportion of 59.9% (n=91) delivered through caesarean delivery. The positive history of diabetes in a family, previous positive history of gestational diabetes and positive previous adverse obstetric history increased chances of caesarean section delivery by more than 3.824 (95% CI = 1.001-14.608, p=0.05), 10.331 (95% CI = 2.464-43.308, p=0.001) and 7.051 (95% CI = 1.577-31.801, 0.01) folds, respectively. Fetal macrosomia incidence was 42.1% (n=64) among women with gestational diabetes. The primary level of education, previous positive history of gestational diabetes and previous positive adverse obstetric history increased the likelihood of fetal macrosomia by more than 6.289 (95% CI = 1.241-31.870, p=0.03), 5.390 (95% CI = 1.498-19.386, p=0.01) and (95% CI = 5.804 1.349-18.423, p=0.02) folds, respectively.

**Conclusion:**

antenatal health care programs and delivery facilities should be strengthened in women with gestational diabetes to improve the risk associated with caesarean delivery and fetal macrosomia.

## Introduction

Gestational diabetes mellitus (GDM) refers to any degree of glucose intolerance at the onset of pregnancy [1]. It is an emerging public health threat to maternal and child health in the world [2]. Globally, the prevalence of GDM varies from 1-28%, depending on the characteristic of the population, diagnostic criteria and screening methods [3]. The prevalence of GDM in Africa is 13.61% and 14.28% in the sub-Sahara region [4]. Africa has a higher GDM prevalence than Europe (5.4%) [5] and Asia (11.5%) [6]. In Kenya, a prevalence of 2.9% has been reported in the western parts of Kenya [7]. Gestational diabetes mellitus (GDM) has several adverse health effects on women and neonates [8]. Women with GDM are likely to suffer from complications such as operative delivery, prolonged labor, pre-eclampsia and the development of type 2 diabetes mellitus in the future [9-11]. The infants are at a risk of macrosomia due to accelerated fetal growth fueled by maternal hyperglycemia, neonatal hypoglycemia and preterm delivery [11,12]. All women with GDM do not deliver through caesarean section. Nevertheless, caesarean delivery is recommended due to several factors such as fetal macrosomia, fetal distress, cervix unfavorable for induction and risk of intrauterine death [13,14]. Several risk factors for caesarean delivery among women with GDM have been reported. These include age above 30 years, overweight or obesity and a family history of diabetes mellitus [15-17]. Fetal macrosomia refers to newborns whose birth weights are greater or equal to 4 kg [18]. It increases the risk of clavicle fracture, shoulder dystocia, and increased need for admission of the neonates in the intensive care unit [18-20]. Research studies have established various risk factors for fetal macrosomia among women with GDM. They include overweight and obesity, previous fetal macrosomia, and history of diabetes mellitus [21-23]. Studies relating to risk factors for adverse health outcomes of GDM are scarce in Kenya. This study was conducted in Nyeri County since it has the highest cases of diabetes in Kenya [24]. Risk factors predisposing to adverse pregnancy outcomes for gestation diabetes mellitus need to be identified to initiate a universal screening before and during pregnancy to identify women at the risk of GDM and ensure the safety of women and newborns. Therefore, this study aimed to determine the risk factors for caesarean delivery and macrosomia among women with GDM in Nyeri County, Kenya.

## Methods

**Study design and setting:** a cross-sectional design was employed in this study. The design described possible related risk factors for caesarean delivery and fetal macrosomia among women with GDM. The study was conducted in four public hospitals (Nyeri County Referral Hospital, Othaya, Karatina and Mukurweini hospitals) in Nyeri County, Kenya.

**Study population:** the study was carried out in Nyeri County, which covers an area of 2361 km^2^. Nyeri County has a population density of 759,164 people, whereby males and females constitute 49% and 51% respectively [25]. The inclusion criteria of this study were women with GDM (18 years and above) and attending the four hospitals in Nyeri County during their pregnancy. The study excluded women with known diabetes and on medications and critically ill women with GDM.

**Variables:** caesarean delivery and fetal macrosomia among women with the GDM were the dependent variables. Independent variables included risk factors such as age group, body mass index (BMI), level of education and occupation, including the medical and obstetric history of women with GDM.

Sample size and sampling: the sample size for the study was calculated using Cochran´s formula [26] to give 152 participants. A simple random sampling technique was used in this study to select women with GDM in each hospital.

Data collection method: data was collected using a structured questionnaire for women with GDM. Other data sources also included antenatal cards and hospital files for birth and delivery records. A family member accompanying the respondents also assisted in providing information during the interview. A pretesting of the research tool was conducted in Kieni East Sub-County in Nyeri County. The structured questionnaires were pretested and the results were cleaned and analyzed accordingly. Research assistants were trained to implement questionnaires to minimize errors during the pretesting stage. Recommendations and suggestions from the pilot study were incorporated in the final questionnaire. Focus group discussions were carried out to complement the questionnaire findings [27].

**Statistical data analysis:** questionnaires data was tabulated in the Microsoft Excel spreadsheet, cleaned and then exported into Statistical Package for Social Sciences (SPSS) software (IBM Corporation, Version 25.0. Armonk, New York, United States of America) for statistical analysis. Descriptive statistics were computed and expressed as frequency and percentage for categorical data or mean ± standard deviation (SD) for continuous data. Univariate analysis was carried out using binary logistic regression to test for an association between a single independent variable and a dependent variable. The significant variables using univariate analysis were subjected to binomial multiple logistic regression to test for independent risk factors between the dependent and independent variables using the ‘enter´ method. The model fitness was tested by Hosmer and Leme´s Show goodness of fit test. Odds ratios were also calculated. The level of significance was set at 95% (p ≤ 0.05). The results were presented in tables.

**Ethical approval:** Kenyatta University graduate school approved this study. Ethical approval to carry out the study was obtained from Kenyatta University Ethics and Research Committee, reference number KU/ERC/APPROVAL/VOL1/18. Authority to conduct the study was sought from the National Commission for Science, Technology and Innovation (NACOSTI), reference number NACOSTI/P/19/1771. In the selected health facility, permission was sought from the respective authority. Informed consent was obtained from the participants before the questionnaires were administered.

## Results

**Characteristics of women with GDM:** as shown in Table 1, a total of 152 women with GDM were enrolled in this study. The women with age group of <25, 25-29, 30-34 and >34 years old had a proportion of 17.1% (n = 26), 25.0% (n = 38), 27.0% (n = 41) and 30.9% (n = 47) respectively. The mean age of gestation women was 30.86 (SD 5.81) years old. The women with BMI of ≥30, 25-29.9 and 18.5-24.9 kg/m^2^ had a proportion of 43.4% (n = 66), 37.5% (n = 57) and 19.1% (n = 29) respectively. Besides, the women with secondary level of education recorded a higher proportion of 52.6% (n =80). Further, a higher proportion of women were unemployed at 66.4% (n = 101).

**Table 1:** socio-demographic characteristics of women with GDM in Nyeri County, Kenya

Variable	Frequency	Percentage
**Age group in years**		
<25	26	17.1
25-29	38	25.0
30-34	41	27.0
>34	47	30.9
BMI (kg/m^2^)		
18.5-24.9	29	19.1
25-29.9	57	37.5
≥30	66	43.4
Level of education		
Primary	43	28.3
Secondary	80	52.6
Tertiary	29	19.1
Occupation		
Employed	14	9.2
Self-employed	37	24.3
Unemployed	101	66.4
Total	152	100

BMI: body mass index; GDM: gestational diabetes mellitus

**Risk factors for caesarian delivery among women with GDM:** in this study, a higher proportion of women with GDM delivered through caesarean section at 59.9% (n = 91). As presented in Table 2, the univariate analysis showed that the women with the age groups of 30-34 and greater than 34 years old, BMI of ≥30 kg/m^2^, positive history of diabetes in the family, previous positive history of GDM and previous positive obstetric history were risk factors for caesarean delivery among women with GDM in Nyeri, County, Kenya (p≤0.05).

**Table 2:** binary and binomial multiple logistic analyses of risk factors for caesarean delivery in women with GDM in Nyeri County, Kenya

	Binary logistic regression analysis			Binomial multiple logistic regression analysis		
Variables	Odd ratio	95% CI	p value	Odd ratio	95% CI	p value
**Age group in years**						
<25	Ref.			Ref.		
25-29	1.889	0.676-5.281	0.23	0.863	0.110-6.764	0.89
30-34	4.068	1.435-11.532	0.01	1.159	0.143-9.412	0.89
>34	5.509	1.946-15.595	0.001	0.486	0.051-4.596	0.53
**BMI (kg/m^2^)**						
18.5-24.9	Ref.			Ref.		
25-29.9	2.432	0.962-6.148	0.06	1.230	0.194-7.779	0.83
≥30	5.476	2.131-14.073	<0.001	1.559	0.234-10.372	0.65
**Level of education**						
Primary	2.473	0.931-6.567	0.07			
Secondary	1.526	0.650-3.583	0.33			
Tertiary	Ref.					
**Occupation**						
Employed	Ref.					
Self-employed	1.467	0.246-5.049	0.54			
Unemployed	1.590	0.518-4.880	0.42			
**Family diabetes history**						
Yes	14.915	6.272-35.465	<0.001	3.824	1.001-14.608	0.05
No	Ref.			Ref.		
**Previous GDM history**						
Yes	43.736	13.907-137.907	<0.001	10.331	2.464-43.308	0.001
No	Ref.			Ref		
**Previous adverse obstetric history**						
Yes	38.187	12.247-119.072	<0.001	7.051	1.577-31.801	0.01
No	Ref.			Ref.		

BMI: body mass index; CI: confidence interval; Ref.: reference; GDM: gestational diabetes mellitus

As presented in Table 2, the multiple logistic regression of the variables that were significant using univariate logistic regression revealed that the positive history of diabetes in a family, positive previous history of gestational diabetes and previous positive adverse obstetric history were independent risk factors for caesarean delivery among women with GDM in Nyeri, County, Kenya (p≤0.05). The positive history of diabetes in a family, positive previous history of gestational diabetes and previous positive adverse obstetric history increased the likelihood of caesarean delivery by more than 3.824 (95% CI = 1.001-14.608, p = 0.05), 10.331 (95% CI = 2.464-43.308, p = 0.001) and 7.051 (95% CI = 1.577-31.801, p = 0.01) folds respectively.

**Risk factors for fetal macrosomia among women with GDM:** in this study, a proportion of 42.1% (n = 64) of women with GDM delivered neonates who were macrosomia. Using univariate analysis, the women with age groups of 30-34 and greater than 34 years, body mass index (BMI) of 25-29.9 and greater or equal to 30 kg/m^2^, primary level of education, positive history of diabetes in a family, previous positive obstetric history were risk factors for fetal macrosomia among women with GDM (p≤0.05; Table 3).

**Table 3:** binary and binomial multiple logistic analyses of risk factors fetal macrosomia in women with GDM in Nyeri County, Kenya

	Binary logistic regression analysis			Binomial multiple logistic regression analysis		
Variables	Odd ratio	95% CI	p value	Odd ratio	95% CI	p value
**Age group in years**						
<25	Ref.			Ref		
25-29	2.860	0.812-10.069	0.10	0.337	0.043-2.640	0.30
30-34	7.028	2.052-24.068	0.002	0.924	0.125-6.842	0.94
>34	5.739	1.713-19.230	0.01	0.282	0.037-2.162	0.22
**BMI (kg/m^2^)**						
18.5-24.9	Ref.			Ref		
25-29.9	3.017	1.003-9.075	0.05	0.517	0.086-3.121	0.47
≥30	6.124	2.081-18.019	0.001	0.416	0.064-2.701	0.36
**Level of education**						
Primary	3.646	1.321-10.060	0.01	6.289	1.241-31.870	0.03
Secondary	1.661	1.655-4.210	0.29	2.065	0.504-8.457	0.31
Tertiary	Ref.			Ref		
**Occupation**						
Employed	Ref.					
Self-employed	1.096	0.305-3.938	0.89			
Unemployed	1.446	0.453-4621	0.53			
**Family diabetes history**						
Yes	10.185	3.918-26.479	<0.001	2.989	0.732-12.206	0.13
No	Ref.			Ref		
**Previous GDM history**						
Yes	15.053	6.405-35.374	<0.001	5.390	1.498-19.386	0.01
No	Ref.			Ref		
**Previous adverse obstetric history**						
Yes	14.424	6.224-33.43	<0.001	5.804	1.349-18.423	0.02
No	Ref.			Ref		

BMI: body mass index; CI: confidence interval; Ref.: reference; GDM: gestational diabetes mellitus

As shown in Table 3, the results of multiple logistic regression of variables that were significant using univariate analysis showed that the primary level of education, positive previous history of gestational diabetes and positive previous adverse obstetric history were independent risk factors for fetal macrosomia among women with GDM in Nyeri County, Kenya (p≤0.05). The women with primary level of education, previous positive history gestational diabetes and previous positive adverse obstetric history increased fetal macrosomia by more than 6.289 (95% CI = 1.241-31.870, p = 0.03), 5.390 (95% CI = 1.498-19.386, p = 0.01) and (95% CI = 5.804 1.349-18.423, p = 0.02) folds respectively.

## Discussion

This study assessed the risk factors for caesarean delivery as well as macrosomia among women with GDM in Nyeri County, Kenya. Adverse pregnancy outcomes have been consistently reported at higher rates in women diagnosed with gestational diabetes mellitus [28,29]. Women with GDM have a higher risk of developing pre-eclampsia and deliver through caesarean delivery [30]. The fetal and neonatal complications of GDM include stillbirth, intrauterine death, fetal macrosomia, shoulder dystocia and increased neonatal intensive care unit admissions [10,31,32]. This study found that diabetes in a family, previous positive history of GDM and positive previous adverse obstetric history were independent risk factors for caesarean delivery. Further, the primary level of education, previous positive history of GDM and previous positive adverse obstetric history were independent risk factors for fetal macrosomia.

Caesarean section is one of the adverse health outcomes associated with GDM in women due to fetal macrosomia [33]. This study observed that the positive history of diabetes in the family was an independent risk for caesarean delivery among women with GDM in Nyeri County, Kenya. The odd ratio of caesarean deliveries was 3.824 times greater in women who had a positive history of diabetes in the family as opposed to those who never had a history of diabetes in the family. This finding was consistent with a similar study carried out by Levy [15] that reported a positive history of diabetes in the family as a risk factor for caesarean delivery among gestational diabetes in Soroka University Medical Center, Israel.

This study also revealed that the positive previous history of GDM was an independent risk factor for caesarean delivery among women with gestational diabetes in Nyeri County, Kenya. The positive history of previous gestational diabetes mellitus increased caesarean deliveries by more than 10.331 folds. This finding agreed with a study carried out by Paramsothy [34] that found the positive history of gestational diabetes as a risk factor for caesarean delivery among women with gestational diabetes mellitus in Washington, United State of America.

This study also observed that previous positive adverse obstetric history was a risk factor for caesarean delivery among women with gestational diabetes mellitus in Nyeri County, Kenya. The caesarean deliveries were likely to increase by more than 7.051 folds for women with positive adverse obstetric history. Some of the previous adverse obstetric histories reported in this study included stillbirth, miscarriage, pre-eclampsia, gestation hypertension, previous caesarean delivery, previous macrosomia, postpartum hemorrhage and premature birth. A study on the risk factors for caesarean delivery following labor induction at a tertiary hospital in North Tanzania: a retrospective cohort study also reported that positive adverse obstetric history was a risk factor for caesarean delivery [35].

The study also determined the risk for fetal macrosomia among women with gestational diabetes in Nyeri County, Kenya. Macrosomia refers to excessive intrauterine fetal growth or birth weight greater or equal to 4 kg, regardless of the gestation age [18]. Macrosomia occurs due to accelerated fetal growth fueled by maternal hyperglycemia [36]. It is a complication linked with increased risks for vaginal lacerations, caesarean delivery, postpartum hemorrhage and need for blood transfusion in mothers [18,20,21]. The fetus may be at risk of shoulder dystocia, brachial plexus palsy, asphyxia, intracranial hemorrhage, hypoglycemia and prolonged stay at the neonatal intensive care unit [18-20,37,38].

In this study, 42.1% of the newborns delivered by women with GDM were macrosomia. The primary level of education was an independent risk factor for fetal macrosomia among women with GDM in Nyeri County, Kenya. The odd ratio of delivering a macrosomia newborn was 6.289 times greater for women who had a primary level of education as opposed to those who had a tertiary level of education. This could be attributed to the limited knowledge on how to manage gestational diabetes mellitus among women who had a primary level of education [39].

The previous positive history of gestational diabetes mellitus was also an independent risk factor for fetal macrosomia among women with GDM in Nyeri County, Kenya. The odds of delivering macrosomia newborns was 5.390 times greater in women with a positive history of GDM than those who never had previous GDM. This finding agreed with a study by Mardani [21] that reported a positive history of GDM as a risk factor for fetal macrosomia among pregnant women with GDM in Asalian Hospital of Khorramabad, Iran.

This study also revealed that positive previous adverse obstetric history among women with GDM was an independent risk factor for fetal macrosomia in Nyeri County, Kenya. The positive previous adverse obstetric history could increase the likelihood of delivering macrosomia newborns by more than 5.390 folds. A study by Bukelskiene [22] also reported that positive previous adverse obstetric history was a risk factor for macrosomia among women with gestational diabetes at Vilnius University Hospital, Santariskiu Klinikos, Lithuania. Also, a study by Mohammadbeigi [40] revealed that positive previous adverse obstetric history was a risk factor for macrosomia in consecutive births occurring in private and public hospitals of Shiraz, Iran.

This study recommends that the gestational diabetic women with a positive history of diabetes in the family, previous positive history of gestational diabetes and positive previous adverse obstetric history should be provided with all delivery facilities and care services to prevent as well as reduce adverse maternal and neonates outcomes that may arise due to caesarean delivery. Also, the gestational diabetic women with a primary level of education, previous positive history of gestational diabetes mellitus, and positive previous adverse obstetric history should attend all of their antenatal care programs so that health professionals can advise them accordingly on how to avoid maternal and fetal complications that may arise due to fetal macrosomia.

**Limitation:** the limitation of this study included recall bias by the participants since it relied on existing medical records and the subject´s recall. This limitation was minimized by retrieving and relating some information from the participants´ medical records and information obtained from the questionnaires.

**Availability of data and materials:** materials described in the manuscript, including all relevant raw data, will be freely available to any scientist wishing to use them for non-commercial purposes without breaching participant confidentiality. The datasets used and/or analyzed during the current study are available from the corresponding author on reasonable request.

## Conclusion

The study concluded that the positive history of diabetes in the family, previous positive history of gestational diabetes and positive previous adverse obstetric history are independent risk factors for caesarean delivery among women with GDM in Nyeri County, Kenya. On the other hand, the independent risk factors for fetal macrosomia were the primary level of education, previous positive history of gestational diabetes mellitus, and positive previous adverse obstetric history among women with GDM in Nyeri County, Kenya

### What is known about this topic

- Gestational diabetes mellitus has adverse health complications in women with GDM and their neonates;
- Women with GDM are likely to suffer from complications such as operative delivery (caesarean delivery), while neonates are at risk of macrosomia

### What this study adds

- The study identified caesarean delivery and macrosomia risk factors among women with GDM;
- The findings of this study are of importance to the stakeholders in healthcare for making strategies concerning maternal and neonatal health among women with GDM.
